# Cochlear implant as an option for unilateral deafness rehabilitation

**DOI:** 10.1590/2317-1782/e20250415en

**Published:** 2026-06-15

**Authors:** Tatiana Rocha Silva, Thiago Motta Oliveira, Camila Maria Bernardes da Silva, Cíntia Santos Silva Machado, Fernanda Abalen Martins Dias

**Affiliations:** 1 Serviço de Atenção à Saúde Auditiva, Pontifícia Universidade Católica de Minas Gerais – PUC Minas - Belo Horizonte (MG), Brasil.; 2 Departamento de Fonoaudiologia, Pontifícia Universidade Católica de Minas Gerais – PUC Minas - Belo Horizonte (MG), Brasil.

Law no. 14,768/2023^(1)^ defines a person with hearing impairment as one who presents with total unilateral or partial or total bilateral hearing limitation and guarantees the rights and benefits of affected individuals. It aims to promote inclusion and equal conditions for people with unilateral or bilateral hearing impairment.

However, per the general guidelines for specialized care for people with hearing impairment under Brazil’s Unified Health System (Sistema Único de Saúde [SUS], Ordinance GM/MS No. 2776/2014^(2)^), cochlear implants (CIs) are indicated for auditory habilitation and rehabilitation of individuals with severe to profound bilateral sensorineural hearing loss (HL). According to these guidelines, CIs are not indicated for unilateral HL (UHL).

The auditory effects of UHL include impairments in the head shadow effect, binaural squelch, and binaural summation. The head shadow effect occurs during everyday listening conditions, when speech and noise are spatially separated, and unlike binaural summation and squelch, it does not require higher-level cortical processing. Similar to the head shadow effect, the binaural squelch occurs when speech and noise are spatially separated and the two ears receive different stimuli. Unlike the head shadow effect, binaural squelch requires central auditory processing, integrating the signal from each ear separately at the level of the auditory cortex to improve audibility. Binaural summation is also an effect of central auditory processing of binaural hearing; however, unlike binaural squelch, which relies on the variation of the signal-to-noise ratio between the ears, it occurs when both ears are presented with a similar signal^(3)^.

Sound localization also requires binaural hearing and is impaired in patients with UHL. It depends on interaural time and intensity differences. For low-frequency sounds, the binaural auditory system relies primarily on phase delays caused by interaural time differences (i.e., the difference in the arrival time of a sound at one ear compared to that at the other). For high frequencies, localization is because of interaural intensity differences arising from the head shadow effect^(4)^.

Therefore, UHL may not only disrupt the central auditory processing strategies and impair auditory function but may also be severely detrimental to the listener's quality of life. In children with UHL, the learning process is impaired compared to that of their peers with normal hearing, resulting in issues such as higher rates of grade retention, a higher incidence of individualized educational support programs, and a greater need for educational resources and attention in the classroom. Furthermore, many of these children are diagnosed with attention deficit and hyperactivity or learning disabilities, because UHL results in the affected individual having to concentrate more to listen.

Contralateral Routing of Signals systems and bone-anchored hearing systems are treatment options for UHL but offer limited benefit in speech recognition in noise. Specifically, these devices improve the head shadow effect, but do not improve the binaural summation, since the sound is "routed" to the better-hearing cochlea and the patient experiences pseudo-binaural hearing. However, CI provides a real acoustic sensation through electrical stimulation to the affected side, so the auditory system can effectively combine these electrical signals with acoustic hearing in the contralateral ear. Thus, patients with implants could also benefit from the summation effect^(5,6)^.

To date, it is unknown whether the critical period of brain plasticity is the same for individuals with bilateral HL and UHL, or whether the ear with normal hearing is able to sustain bilateral cortical maturation and prolong the critical period. If this critical period is temporarily prolonged, CIs can be successful in children with UHL. However, the duration of this extension is currently unknown. If the critical period is prolonged indefinitely, CIs may also be beneficial in adults with UHL^(7,8)^.

A revision and update of the CI candidacy criteria are urgently needed, both in SUS (the Brazilian public health system) and in private healthcare networks, and the inclusion of UHL is important. The Brazilian Society of Otology and the Brazilian Association of Otorhinolaryngology and Cervicofacial Surgery have already established CI candidacy criteria for adults who present with severe or profound UHL or bilateral pre- and post-lingual sensorineural HL. In the third edition of the Audiology Treatise, the Brazilian Academy of Audiology presents considerations on CI candidacy in UHL ^(9)^. In countries such as Germany, the United States, and Australia, UHL is already a formal criterion for CI candidacy. Furthermore, indications for CI in the United States were expanded in 2019 to include individuals with UHL. In this context, in 2022, the American Cochlear Implant Alliance Task Force Guidelines for Clinical Assessment and Management of Adult Cochlear Implantation for Single-Sided Deafness were established^(10)^.

Some indications for the use of CI in UHL can be found in the literature, such as in the late stage of Ménière's disease, in cases of risk to the only hearing ear, and in children with progressive HL^(11-13)^. The criteria for CI are evolving, and children with UHL or asymmetric HL, who were traditionally not considered candidates for CI, are now being individually assessed and considered as such.

To determine whether patients with UHL are eligible to receive CIs, it is necessary to know the etiology of the HL, the hearing conditions in the contralateral ear, and whether there is any threat to the contralateral (better-hearing) ear. Anticipated future HL in the contralateral (better-hearing) ear is among the most compelling indications for CI in patients with UHL.

The decision to implant a child with UHL raises a unique set of questions and presents several challenges, in addition to regulatory hurdles. It is unknown which children actually develop cognitive and behavioral problems related to UHL. Further, there is no measure to identify the "at-risk" group that would benefit from CI, and waiting to determine the need increases the duration of deafness, which may affect CI outcomes.

Consistent studies following up on individuals with implants are lacking. However, given the options currently available, CIs may be an excellent treatment option for patients with UHL and tinnitus and for patients who have not benefited from other forms of treatment^(14)^.

The significant clinical heterogeneity among studies evaluating CI in UHL makes it difficult to conduct a robust analysis with homogeneous groups. Studies have reported great variation with regard to the duration and onset of HL, and some do not mention the duration of sound deprivation. Further, there is disagreement regarding the follow-up, especially concerning the tests and parameters used to evaluate the outcomes. Therefore, it is of utmost importance to conduct consistent studies on this topic to consolidate CI indication protocols as a viable alternative for the treatment of UHL in adults and children.

Establishing laws and guidelines is essential to ensure the efficiency, transparency, and legality of actions pertaining to CI. Therefore, the general guidelines regarding specialized care for people with hearing impairment in SUS – Ordinance GM/MS No. 2776/2014^(2)^ must be reviewed and updated, ensuring that Law No. 14,768/2023^(1)^, which aims to promote inclusion and equal conditions for individuals with UHL or bilateral hearing impairment, extends not only to rights and benefits but also to the rehabilitation of auditory abilities.
