# Supplementary material for: Cochlear implant as an option for unilateral deafness rehabilitation
Source: Codas. 2026 Jun 15;38(4):e20250415. doi: 10.1590/2317-1782/e20250415en (PMC13322489; doi:10.1590/2317-1782/e20250415en)
Supplement: Translation

# Translation: Implante coclear como opção para a reabilitação da surdez unilateral

**DOI:** 10.1590/2317-1782/e20250415pt

**Authors:** Tatiana Rocha Silva, Thiago Motta Oliveira, Camila Maria Bernardes da Silva, Cíntia Santos Silva Machado, Fernanda Abalen Martins Dias

**Affiliations:** 1 Serviço de Atenção à Saúde Auditiva, Pontifícia Universidade Católica de Minas Gerais – PUC Minas - Belo Horizonte (MG), Brasil.; 2 Departamento de Fonoaudiologia, Pontifícia Universidade Católica de Minas Gerais – PUC Minas - Belo Horizonte (MG), Brasil.

A Lei nº 14.768 / 2023^(1)^ considera como pessoa com deficiência auditiva quem apresenta limitação unilateral total ou bilateral parcial ou total, garantindo direitos e benefícios. A Lei visa promover a inclusão e a igualdade de condições para pessoas com deficiência auditiva, seja esta unilateral ou bilateral.

Entretanto, as diretrizes gerais para a atenção especializada às pessoas com deficiência auditiva no Sistema Único de Saúde (SUS) – Portaria GM/MS Nº 2776 / 2014^(2)^, estabelece que o uso de implante coclear (IC) está indicado para habilitação e reabilitação auditiva de pessoas que apresentam perda auditiva sensorioneural bilateral, de grau severo a profundo. Nessas diretrizes, o IC não é indicado para a perda auditiva unilateral.

Os efeitos auditivos da surdez unilateral (SU) incluem prejuízos no efeito sombra da cabeça, no silenciador e na somação binaural. O efeito sombra da cabeça ocorre durante as condições de audição cotidianas, quando a fala e o ruído são espacialmente separados e, diferentemente da somação e do silenciador, não requerem processamento cortical de nível superior. Assim como o efeito sombra da cabeça, o silenciador binaural ocorre quando a fala e o ruído são espacialmente separados e os dois ouvidos recebem estímulos diferentes. Ao contrário do efeito sombra da cabeça, o silenciador binaural requer processamento auditivo central, integrando o sinal de cada ouvido isoladamente ao nível do córtex auditivo para melhorar a audibilidade. A somação binaural também é um efeito do processamento auditivo central da audição binaural, mas, ao contrário do silenciador, que depende da variação da relação sinal ruído entre os ouvidos, a somação ocorre quando ambos os ouvidos são apresentados a um sinal semelhante^(3)^.

A localização sonora também requer audição binaural e é prejudicada em pacientes com SU. A localização sonora depende das diferenças de tempo interaural e das diferenças de intensidade interaural. Para sons de baixa frequência, o sistema auditivo binaural depende principalmente de atrasos de fase causados por diferenças de tempo interaural (ou seja, a diferença no tempo de chegada de um som em um ouvido em comparação ao outro). Para altas frequências, a localização decorre, principalmente, das diferenças de intensidade interaural que surgem do efeito de sombra da cabeça^(4)^.

Portanto, a SU, pode não apenas interromper as estratégias de processamento auditivo central e prejudicar a função auditiva, mas também ser gravemente prejudicial à qualidade de vida do ouvinte. Em crianças com SU, o processo de aprendizagem é prejudicado em comparação com o de seus pares com audição normal, incluindo maiores taxas de reprovação, maior incidência em programas de acompanhamento educacional individualizado e maior necessidade de recursos educacionais e atenção em sala de aula. Além disso, muitas dessas crianças são diagnosticadas com déficit de atenção e hiperatividade ou como tendo dificuldades de aprendizagem, uma vez que a SU exige maior concentração para ouvir.

O sistema de Roteamento Contralateral do Sinal (CROS) e as Próteses Auditivas com Ancoramento Ósseo (PAAO) são possibilidades terapêuticas nos casos de SU, porém oferecem benefício limitado no reconhecimento de fala no ruído. De fato, esses dispositivos melhoram o efeito sombra da cabeça, mas não melhoram o efeito de somação das vias auditivas, uma vez que, o som é “roteado” para a melhor cóclea e o paciente tem uma pseudobinauralidade. O IC, no entanto, oferece uma real sensação acústica através de uma estimulação elétrica para o lado afetado, assim, o sistema auditivo pode efetivamente combinar este sinal elétrico com audição acústica na orelha oposta. Com isso, os pacientes implantados também poderiam se beneficiar do efeito de somação^(5,6)^.

Até o momento, não se sabe se o período crítico de plasticidade cerebral é o mesmo para indivíduos com perda auditiva bilateral e unilateral ou se o ouvido com audição normal é capaz de sustentar a maturação cortical bilateral e prolongar o período crítico. Se esse período crítico for prolongado temporariamente, o IC em crianças com SU pode ser bem-sucedido. No entanto, a duração desse prolongamento é atualmente desconhecida. Se o período crítico for prolongado indefinidamente, o IC em adultos com perda auditiva unilateral também pode ser benéfico^(7,8)^.

É urgente uma revisão e atualização dos critérios de indicação ao IC, tanto no SUS quanto na rede de assistência privada. É importante a inclusão da SU. A Sociedade Brasileira de Otologia (SOB) e a Associação Brasileira de Otorrinolaringologia e Cirurgia Cérvico-Facial (ABORL-CCF) já estabeleceram critérios de indicação do IC para adultos que apresentam perda auditiva sensorioneural pré-lingual e pós-lingual de grau severo ou profundo unilateral ou bilateral. A Academia Brasileira de Audiologia (ABA) na terceira edição do Tratado de Audiologia apresenta considerações da indicação do IC na SU^(9)^. Em países como Alemanha, Estados Unidos e Austrália a SU já é um critério formal de indicação para o IC. As indicações para IC nos Estados Unidos foram ampliadas em 2019 para incluir indivíduos com SU. Nesse contexto, em 2022 foram estabelecidas as Diretrizes da Força Tarefa da Aliança Americana de Implante Coclear para Avaliação Clínica e Manejo do Implante Coclear em Adultos com Surdez Unilateral^(10)^.

Na literatura existem algumas indicações relatadas do uso do IC em SU, como na fase tardia da Doença de Ménière, em casos de risco ao único ouvido bom e em crianças com perda auditiva progressiva^(11-13)^. Os critérios de indicação para o IC estão evoluindo e crianças com SU ou perda auditiva assimétrica que tradicionalmente não eram consideradas candidatas ao IC já estão sendo avaliadas individualmente e consideradas candidatas ao IC.

Para determinar a elegibilidade para pacientes com SU, é necessário conhecer a etiologia da perda auditiva, as condições da audição na orelha contralateral e se há alguma ameaça à orelha contralateral (com melhor audição). A antecipação de futura perda auditiva na orelha contralateral (melhor audição) do paciente está entre as indicações mais persuasivas para IC em pacientes com SU.

A decisão de implantar uma criança com SU levanta um conjunto peculiar de questões e apresenta vários desafios, além de obstáculos regulatórios. Não se sabe quais crianças realmente desenvolvem problemas cognitivos e comportamentais relacionados com a SU. Não há uma medida para a identificação do grupo “de risco” que se beneficiaria do IC. Aguardar para determinar a necessidade aumentará o tempo da surdez, o que pode ter impacto sobre os resultados com o IC.

Faltam estudos consistentes e que acompanhem os indivíduos implantados, entretanto diante das opções disponíveis atualmente, o IC pode ser uma ótima opção de tratamento para pacientes com SU nos casos de presença de zumbido e para pacientes que não se beneficiaram com outras formas de tratamento^(14)^.

Há uma grande heterogeneidade clínica entre os estudos que avaliam o IC na SU, o que dificulta a realização de uma análise sólida com grupos homogêneos. Os estudos apresentam uma grande variação quanto à duração e início da surdez, em alguns estudos não é mencionado o tempo de privação sonora. Existe divergência em relação ao acompanhamento e principalmente no que se refere aos testes e parâmetros utilizados para avaliar os resultados. É de fundamental importância a realização de estudos consistentes sobre esse tema, a fim de consolidar protocolos de indicação do IC como uma alternativa viável para o tratamento da SU em adultos e crianças.

O estabelecimento de leis e diretrizes é um processo essencial para garantir a eficiência, transparência e legalidade das ações que envolvem o IC. Portanto, é necessário que as diretrizes gerais para a atenção especializada às pessoas com deficiência auditiva no SUS – Portaria GM/MS Nº 2776 / 2014^(2)^, sejam revistas e atualizadas garantindo que a Lei nº 14.768 / 2023^(1)^ que visa promover a inclusão e a igualdade de condições para indivíduos com deficiência auditiva unilateral ou bilateral, seja válida não apenas nos direitos e benefícios, mas também na reabilitação das habilidades auditivas.
